# Arthroscopic Posterior Glenoid Bone Block Using an Adapted Anterior Glenoid Guide and Independent Cortical Button Fixation

**DOI:** 10.1002/atn2.70137

**Published:** 2026-05-25

**Authors:** Pablo Cañete San Pastor, M Inmaculada Prósper Ramos

**Affiliations:** ^1^ Department of Orthopaedic Surgery and Traumatology Hospital de Manises Valencia Spain

## Abstract

Recurrent posterior shoulder instability associated with posterior glenoid bone loss remains challenging. Arthroscopic bone block procedures have gained popularity due to their minimally invasive nature and their ability to restore glenoid concavity while addressing associated intra‐articular pathology. We describe a fully arthroscopic posterior glenoid bone block technique using a tricortical iliac crest autograft fixed with independent cortical buttons from the Glenoid Bone Loss‐Advanced Instability System (Smith & Nephew Andover, MA, USA). A commonly available anterior glenoid guide is used with an adapted posterior application. The technique emphasizes meticulous posterior capsulolabral mobilization, preparation of a bleeding posterior glenoid bone bed, direct visualization for accurate tunnel placement, progressive independent button tensioning, and posterior capsulolabral reconstruction, leaving the graft extra‐articular.

## Graphical abstract

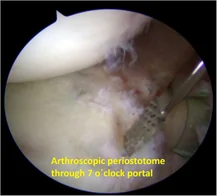

**VIDEO 1:** This video shows a step‐by‐step arthroscopic posterior bone block technique using a tricortical iliac crest autograft for the treatment of persistent posterior right shoulder instability associated with posterior glenoid bone loss. The patient is positioned in the lateral decubitus position with the operative arm under balanced traction. Standard arthroscopic portals are established, including a posterior diagnostic portal, an anterosuperior viewing portal, and an anterior working portal. An additional dedicated 7‐o’ portal is created to allow safe and effective mobilization of the posterior capsulolabral complex. Initial diagnostic arthroscopy confirms the presence of posterior instability and posterior glenoid bone deficiency. A wide and complete detachment of the posterior capsulolabral complex is performed using arthroscopic instruments and a periosteotome introduced through the 7‐o'clock portal. Adequate mobilization of the posterior capsule is essential to create a working space between the capsule and the posterior glenoid rim and to allow subsequent graft placement (Video 1). A traction suture is then placed through the posterior capsulolabral complex using a suture passer introduced through the 7‐o'clock portal. This traction suture retracts the capsule posteriorly and inferiorly, significantly improving visualization and maintaining a consistent working space. Posterior portal dilation is performed, and digital palpation (“finger test”) is used to confirm that the portal size is sufficient to allow atraumatic passage of the bone graft. Under direct visualization from the anterosuperior portal, a specific posterior bone block Smith & Nephew glenoid guide is introduced through the posterior portal. The guide is carefully positioned on the posterior glenoid, and its orientation is adjusted to compensate for the posterior glenoid slope. Two parallel posterior‐to‐anterior glenoid tunnels are drilled using cannulated drills, spaced 1 cm apart and positioned approximately 5 mm medial to the posterior articular surface. Throughout drilling, the distance between the cannulas and the articular cartilage is visually confirmed to avoid cartilage injury or tunnel malposition. Shuttle sutures are passed through the cannulated drills and retrieved through the anterior portal via a small capsular incision. A tricortical iliac crest autograft is harvested and prepared on the back table. Using a specific Smith & Nephew instrumentation clamp, 2 drill holes are created in the graft, reproducing the same spacing and distance from the articular edge as the previously created glenoid tunnels. Cortical buttons are mounted on the graft sutures in preparation for fixation. The graft is introduced through the dilated posterior portal while traction is applied to the shuttle sutures from the anterior portal. A grasper introduced posteriorly assists controlled graft passage and proper orientation. The graft is seated flush against the posterior glenoid surface, and fixation is achieved using 4 cortical buttons (2 posterior and 2 anterior). The anterior buttons are secured on the anterior glenoid cortex, and fixation is completed using Nice knots, providing strong and controlled compression of the graft against the glenoid. Finally, a meticulous posterior capsulolabral reconstruction is performed using all‐suture anchors to restore soft‐tissue stability and proprioception. The capsulolabral complex is repaired in a manner that leaves the bone graft in an extra‐articular position, reducing the risk of intra‐articular hardware prominence and postoperative glenohumeral osteoarthritis. Video content can be viewed at https://doi.org/10.1002/atn2.70137.

Posterior shoulder instability is an uncommon and frequently underdiagnosed condition that represents approximately 2% to 10% of all shoulder instability cases.^1^, ^2^, ^3^ Its clinical presentation is often subtle, with vague symptoms and poorly defined instability events, which complicates diagnosis and delays treatment.^1^, ^2^, ^3^, ^4^ Structural risk factors such as posterior glenoid bone loss, glenoid dysplasia, increased retroversion, and failed previous soft‐tissue repairs have been associated with recurrent posterior instability and inferior outcomes after isolated capsulolabral repair.^4^, ^5^, ^6^, ^7^, ^8^

Posterior bone block procedures have therefore been advocated in cases with significant posterior glenoid deficiency, posterior bony Bankart lesions, glenoid erosion, or failed previous stabilization.^9^, ^10^, ^11^, ^12^ Although traditionally performed through open approaches, arthroscopic bone block techniques have gained popularity due to their minimally invasive nature, superior visualization, ability to treat concomitant intra‐articular pathology, and improved control of graft positioning.^5^, ^6^, ^7^, ^13^, ^14^, ^15^, ^16^, ^17^

Several arthroscopic techniques have been described using screws, suture cerclage constructs, or cortical button fixation.^6^, ^14^, ^15^, ^16^, ^17^, ^18^ Screw fixation, however, has been associated with hardware‐related complications, graft malposition, impingement, pain, osteolysis, and the need for secondary hardware removal.^11^, ^19^, ^20^, ^21^ Conversely, low‐profile cortical button fixation systems allow controlled graft compression, fine‐tuning of graft position, and avoidance of metal prominence.^5^, ^7^, ^16^

The purpose of this Technical Note is to describe an all‐arthroscopic posterior glenoid bone block technique using an adapted use of an anterior glenoid guide and independent cortical button fixation (Glenoid Bone Loss Advanced Instability System, Smith & Nephew, Andover, MA, USA), combined with meticulous posterior capsulolabral reconstruction. This approach leverages surgeons’ familiarity with anterior bone block instrumentation while providing a reproducible, minimally invasive solution for posterior instability.

## SURGICAL TECHNIQUE

### Patient Positioning and Setup

The patient is placed in the lateral decubitus position with the affected shoulder facing upward. The arm is positioned in neutral rotation and suspended with balanced traction using a traction system (approximately 3‐5 kg). Adequate padding is applied to all pressure points (Video 1).

The surgical field is prepared and draped to include both the shoulder and the ipsilateral iliac crest, allowing simultaneous graft harvesting if required.

### Portal Placement and Initial Assessment

A standard posterior portal is established first for diagnostic arthroscopy. An anterosuperior portal is then created under direct visualization and is used as the primary viewing portal throughout most of the procedure. A standard anterior portal is established for suture retrieval and instrumentation.

An accessory posterolateral portal (portal 7) is created under direct visualization and is used to perform posterior capsulolabral mobilization, preparation of the posterior glenoid neck and to place a traction suture (Table 1).

**TABLE 1:** Pearls and Pitfalls

**Pearls**	**Pitfalls**
Achieve complete posterior capsulolabral release before glenoid preparation	Inadequate capsular release may prevent correct graft seating
Use traction sutures to improve visualization and working space	Excessive lateralization increases the risk of osteoarthritis
Reproducing identical tunnel distances on graft and glenoid improves accuracy	Inadequate capsulolabral reconstruction compromises stability
Independent button fixation allows fine‐tuning of graft position	Excessive medialization reduces stabilizing effect
Adjust graft position before final tensioning	Insufficient portal dilation may damage the graft during insertion

### Diagnostic Arthroscopy

A systematic diagnostic arthroscopy is performed to confirm posterior glenoid bone loss, evaluate the condition of the posterior capsulolabral complex, and identify associated intra‐articular pathology. Particular attention is paid to the size and location of the posterior glenoid defect.

### Posterior Capsulolabral Mobilization

A wide and meticulous mobilization of the posterior capsulolabral complex is essential. Viewing from the anterosuperior portal, the posterior labrum and capsule are released from the glenoid rim using a combination of rasps, periosteal elevators, radiofrequency ablation, and shaver devices, introduced through the anterosuperior and accessory portal 7.

**FIGURE 1:**
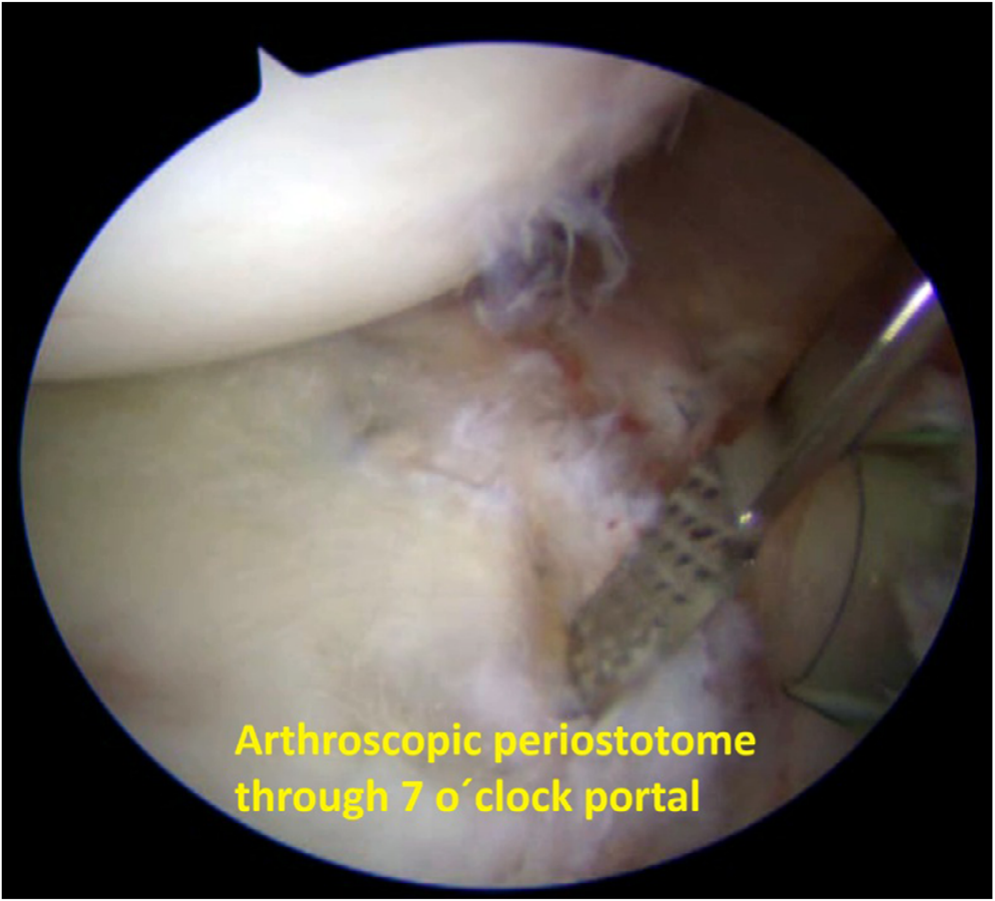
Arthroscopic view of the posterior glenoid of the right shoulder with the patient in lateral decubitus position. Viewing from the anterosuperior portal, a wide detachment of the posterior capsulolabral complex is performed using a periosteotome and arthroscopic instruments introduced through the 7‐o'clock portal. A complete and extensive release of the posterior capsulolabral complex is essential to create an adequate working space and allow correct positioning of the posterior bone graft against the glenoid surface.

The posterior glenoid neck is fully exposed to allow direct visualization of the future bone block position. Complete release is essential to allow posterior translation of the capsule and creation of sufficient working space (Figure 1).

### Glenoid Preparation

The posterior glenoid surface is prepared to obtain a flat, bleeding cancellous bone bed using rasps, a burr, and curettes. Proper preparation of the posterior cortical surface is critical to facilitate graft integration and long‐term stability.

### Traction Suture and Working Space Creation

**FIGURE 2:**
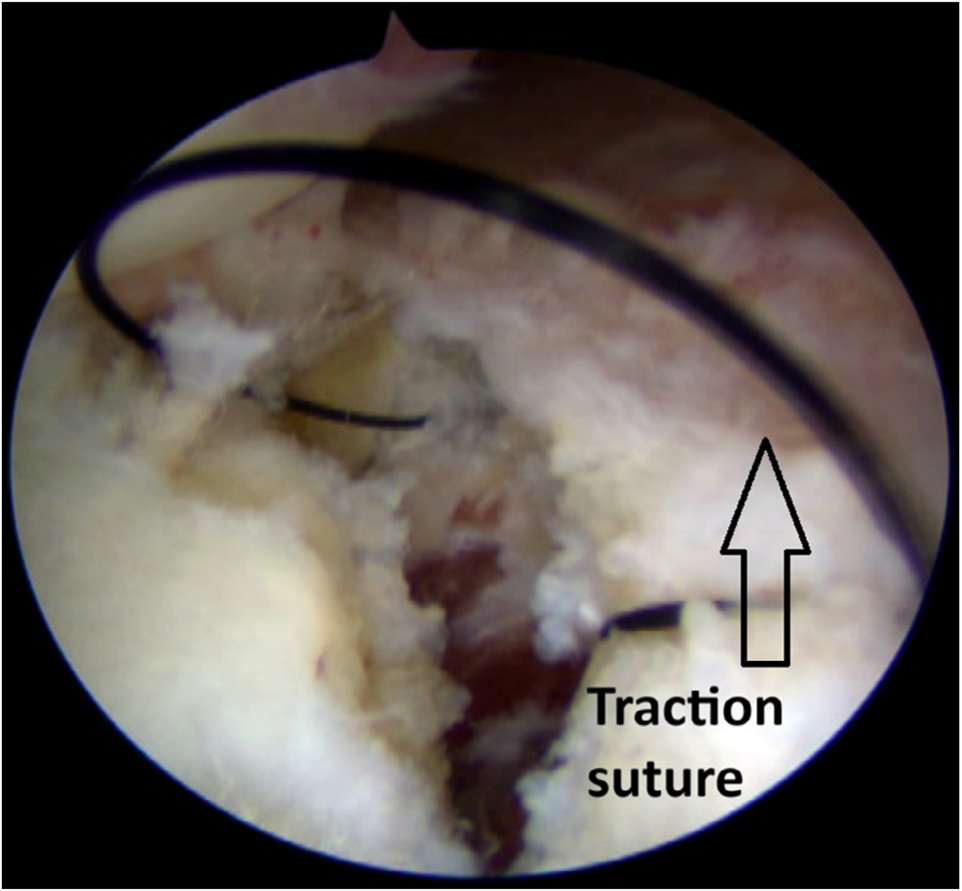
Arthroscopic view of the right shoulder from the anterosuperior portal showing placement of a traction suture through the posterior capsulolabral complex using an AcuPass suture passer introduced through the 7‐o'clock portal. The traction suture is used to retract the posterior capsule away from the glenoid, facilitating visualization, increasing the working space, and protecting soft tissues during subsequent glenoid preparation and graft introduction.

A traction suture is placed through the posterior capsulolabral complex using an AcuPass device (Smith & Nephew, Andover, MA, USA) introduced through portal 7 (Figure 2).

By applying gentle traction on this suture from the posterior portal, the working space between the posterior capsule and the glenoid is significantly increased. This maneuver improves visualization, facilitates glenoid preparation, and creates sufficient space for safe graft introduction.

### Posterior Portal Dilation

**FIGURE 3:**
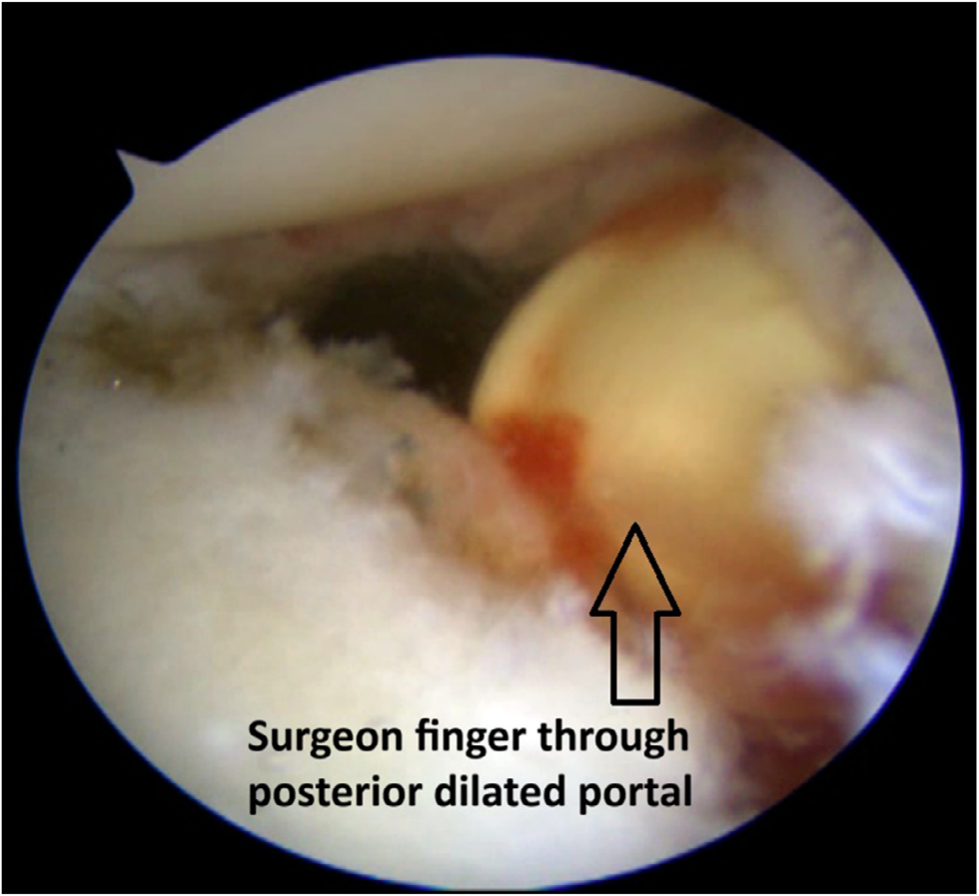
Arthroscopic view of the right shoulder through anterosuperior portal. Posterior portal dilation performed after capsular traction, followed by the “finger test” to confirm that the portal diameter is sufficient to allow atraumatic passage of the iliac crest bone graft. Adequate posterior portal dilation is critical to avoid graft damage, soft‐tissue interposition, or difficulty during graft insertion.

The posterior portal is progressively dilated to allow atraumatic passage of the bone graft. Adequate portal size is confirmed using the “finger test”, in which the surgeon introduces a finger through the posterior portal to ensure sufficient diameter for graft passage without resistance (Figure 3).

### Glenoid Tunnel Drilling

With visualization from the anterosuperior portal, the glenoid guide originally designed for anterior bone block procedures is introduced through the posterior portal. Because posterior glenoid bone loss is less frequent, most surgeons are highly familiar with this guide, which improves comfort and reproducibility.

**FIGURE 4:**
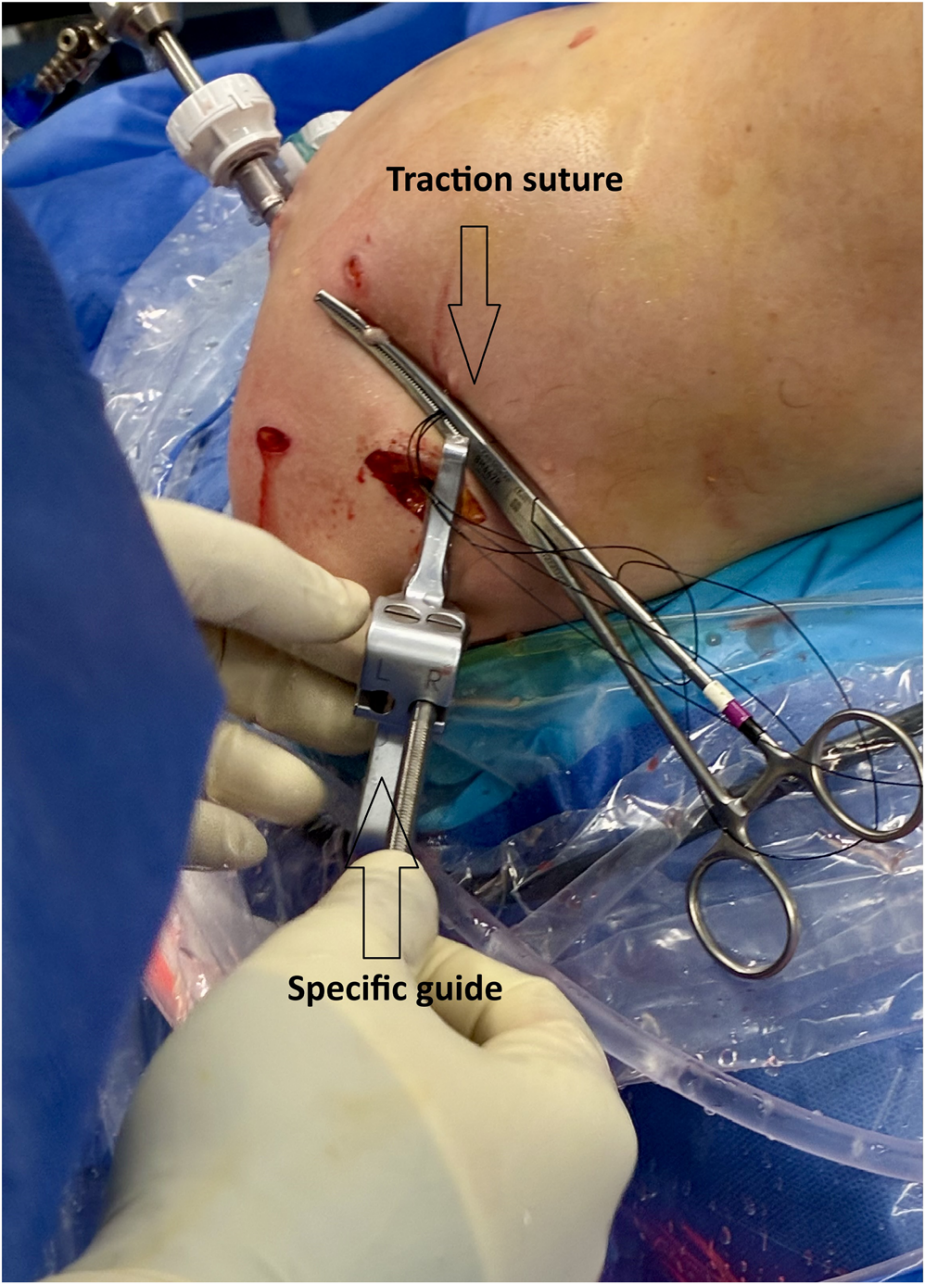
Right shoulder with patitent in lateral decubitus. Introduction of the specific posterior bone block glenoid guide (Smith & Nephew, Andover, MA, USA) through the posterior portal under direct arthroscopic visualization from the anterosuperior portal. The traction suture placed through the posterior capsulolabral complex can be seen exiting the 7‐o'clock portal and secured externally with a clamp, maintaining a consistent working space between the posterior capsule and the posterior glenoid rim.

**FIGURE 5:**
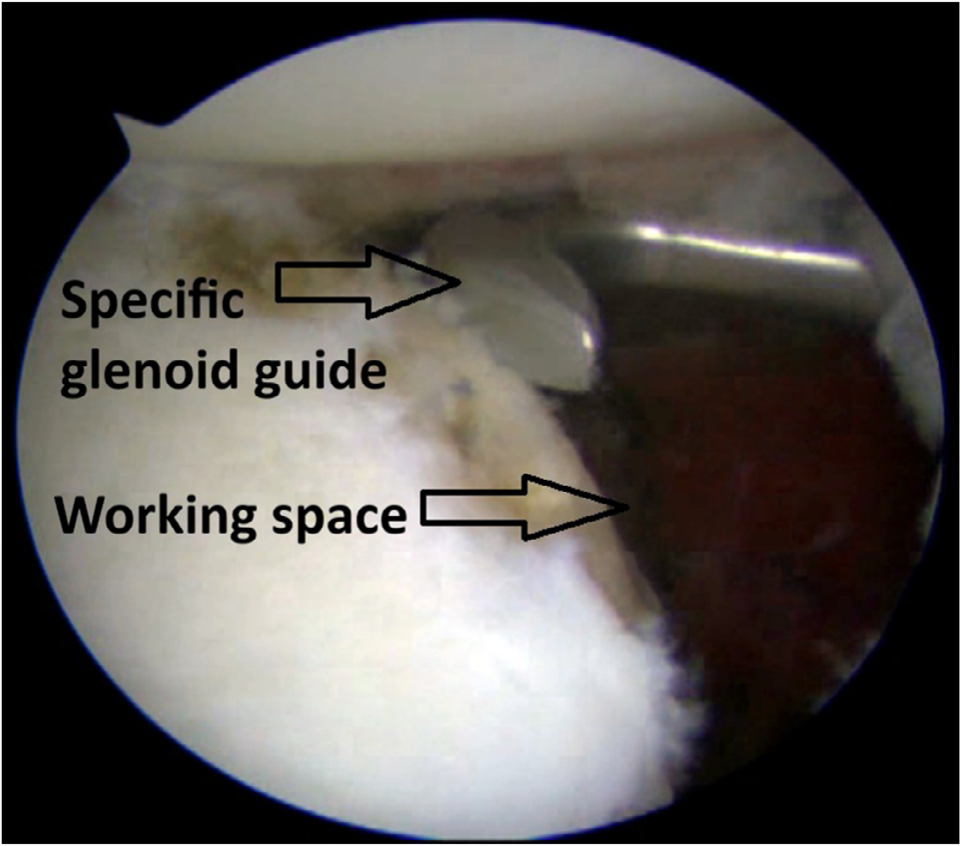
Arthroscopic view of the right shoulder from the anterosuperior portal showing the correct positioning of the posterior bone block glenoid guide (Smith & Nephew, Andover, MA, USA) through the posterior portal. The large working space created by the capsular traction suture is clearly visible, facilitating safe guide placement, accurate tunnel positioning, and reducing the risk of capsular or cartilage injury during drilling.

The guide is slightly elevated and angled to adapt it to the posterior glenoid anatomy. The position of the drill sleeves relative to the posterior articular surface is carefully checked to avoid excessive medialization or lateralization (Figures 4 and 5).

**FIGURE 6:**
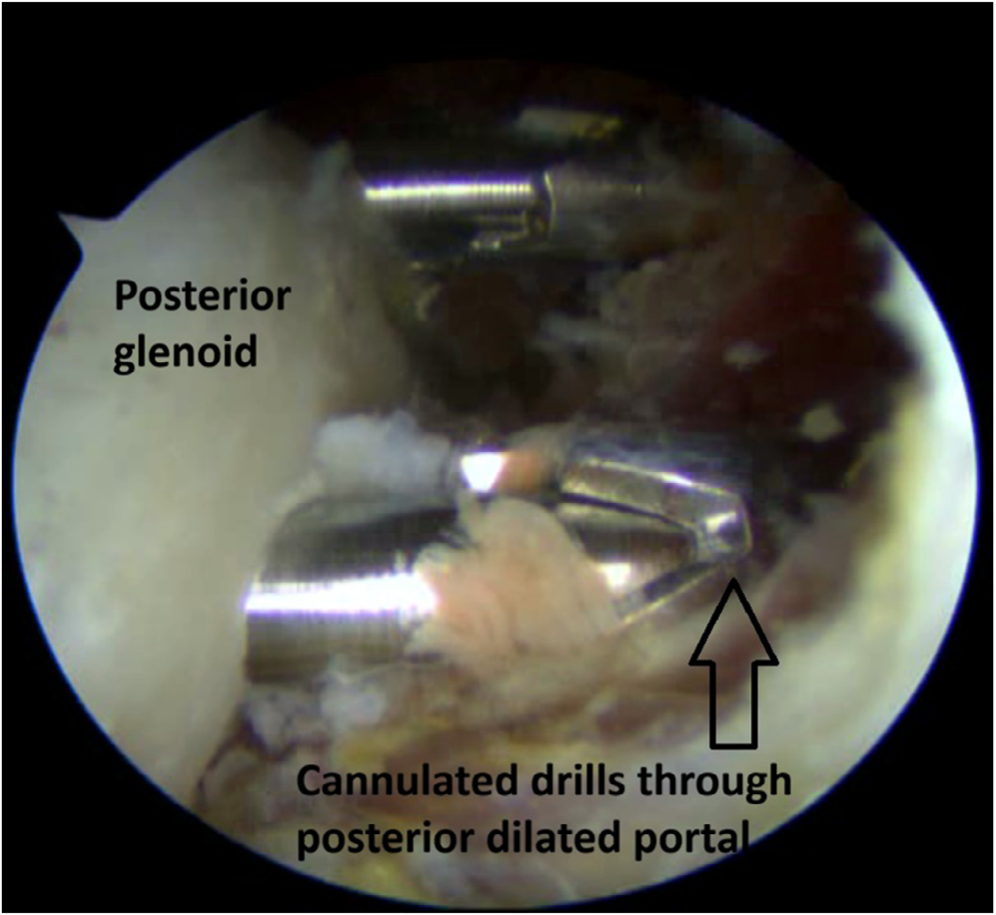
Arthroscopic view of the right shoulder through anterosuperior portal. Drilling of 2 parallel posterior‐to‐anterior glenoid tunnels using the posterior bone block glenoid guide (Smith & Nephew, Andover, MA, USA) introduced through the posterior portal. The tunnels are spaced 1 cm apart and positioned 5 mm medial to the posterior articular surface. Under direct arthroscopic visualization, the distance between the cannulas and the posterior cartilage surface is carefully checked to prevent tunnel misplacement and avoid iatrogenic cartilage injury.

Two parallel glenoid tunnels are drilled from posterior to anterior using cannulated drills, spaced 1 cm apart and approximately 5 mm from the articular surface. These distances exactly reproduce those created previously on the iliac crest graft (Figure 6).

**FIGURE 7:**
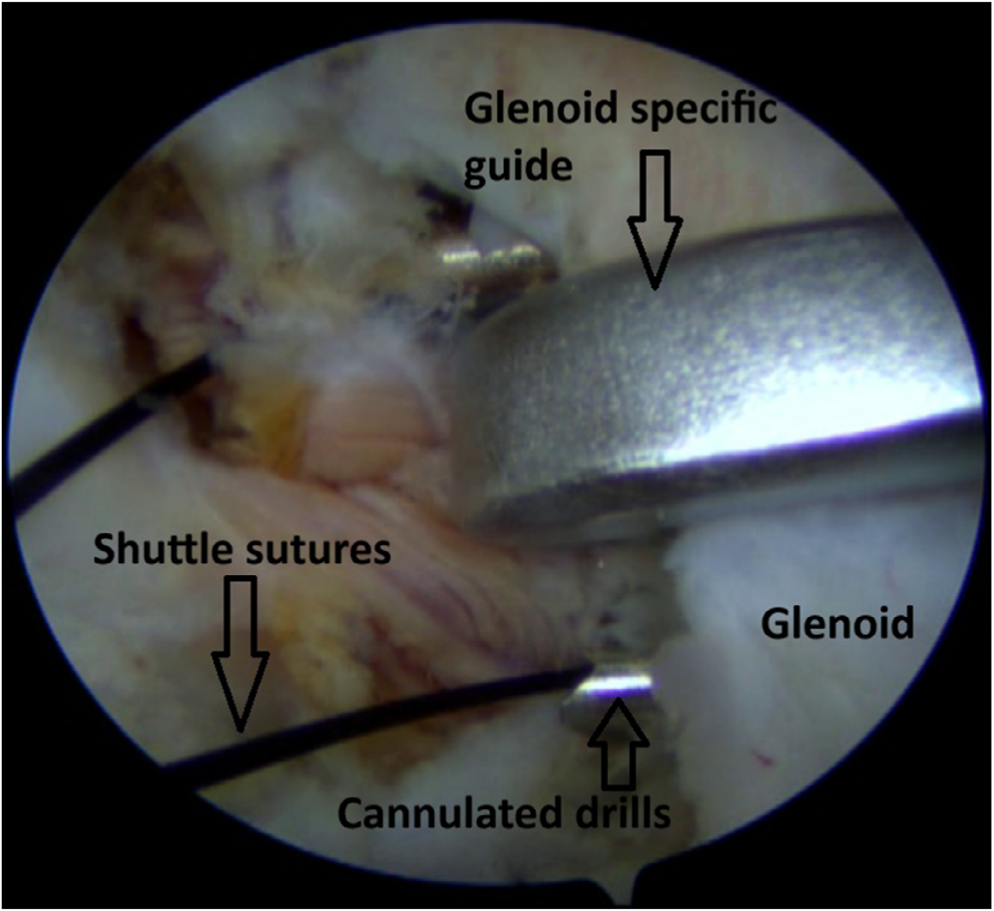
Arthroscopic view of the anterior glenoid of the right shoulder from the anterosuperior portal showing passage of shuttle sutures through the cannulated drills from posterior to anterior. The shuttle sutures are retrieved through the anterior portal after a small capsular incision, allowing controlled graft passage and subsequent fixation from posterior to anterior.

**FIGURE 8:**
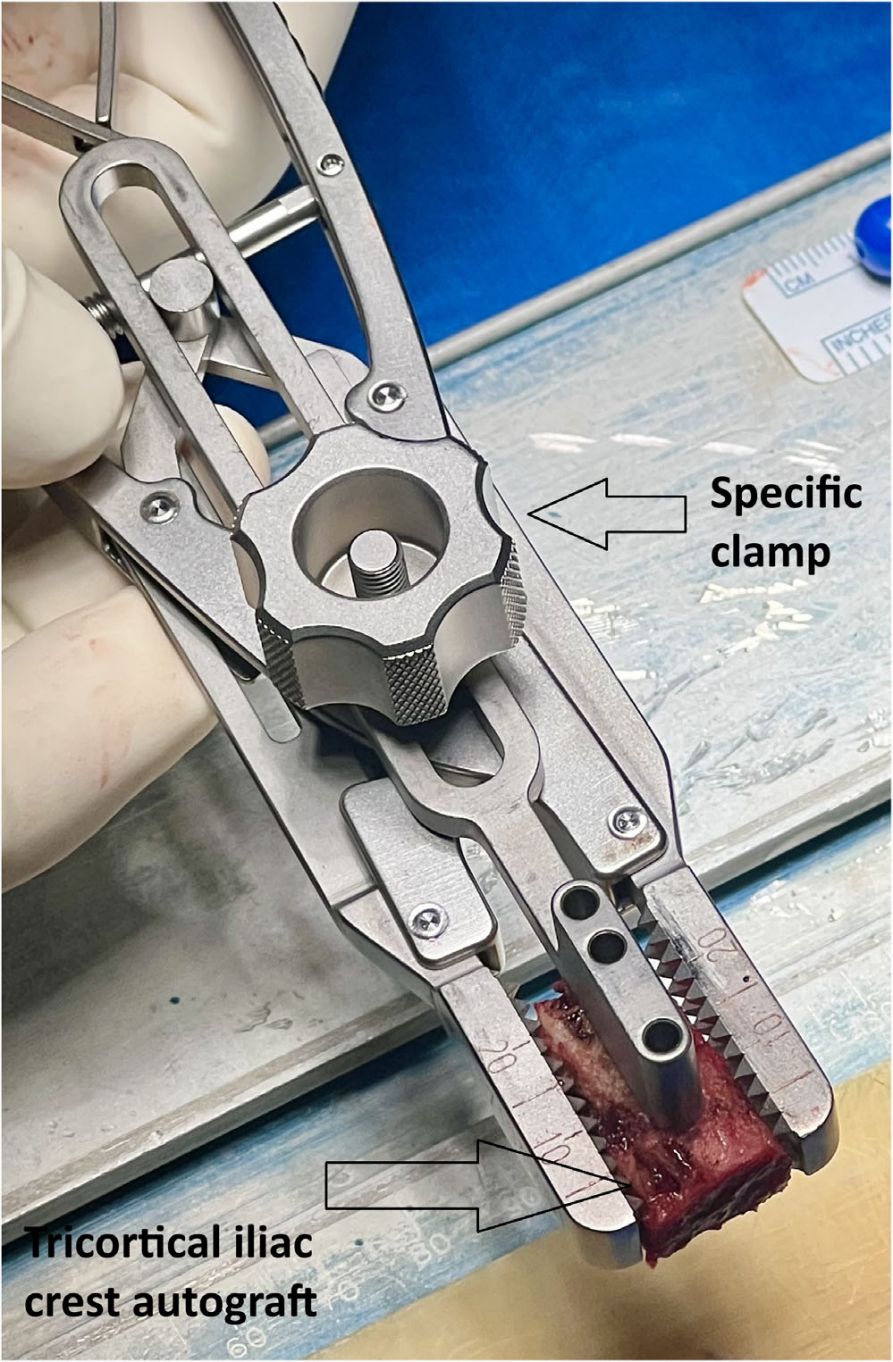
Preparation of a tricortical iliac crest autograft on the back table using the specific Smith & Nephew (Andover, MA, USA) instrumentation clamp. Two drill holes are created in the graft, spaced 1 cm apart and located 5 mm from the graft's articular edge, accurately replicating the tunnel configuration created in the posterior glenoid to ensure precise graft positioning and stable fixation.

Shuttle sutures are passed through the cannulated drills from posterior to anterior (Figures 7 and 8) and retrieved through the anterior portal at the exit point on the anterior glenoid cortex. A small anterior capsulotomy is performed if necessary to facilitate suture retrieval.

### Iliac Crest Graft Harvest and Preparation

A tricortical autograft is harvested from the ipsilateral iliac crest using an oscillating saw and osteotomes. Autologous iliac crest graft is preferred due to its excellent biological integration, unlimited availability, and low donor‐site morbidity in young patients.

Using the specific graft‐holding clamp from the instrumentation set, 2 holes are drilled in the graft 1 cm apart and 5 mm from the articular edge, exactly reproducing the geometry of the glenoid tunnels.

Cortical buttons from the Glenoid Bone Loss Advanced Instability System (Smith & Nephew, Andover, MA, USA) are placed on the cortical surface of the graft, while the cancellous surface is oriented to contact the posterior glenoid surface (Figure 8).

### Graft Introduction

**FIGURE 9:**
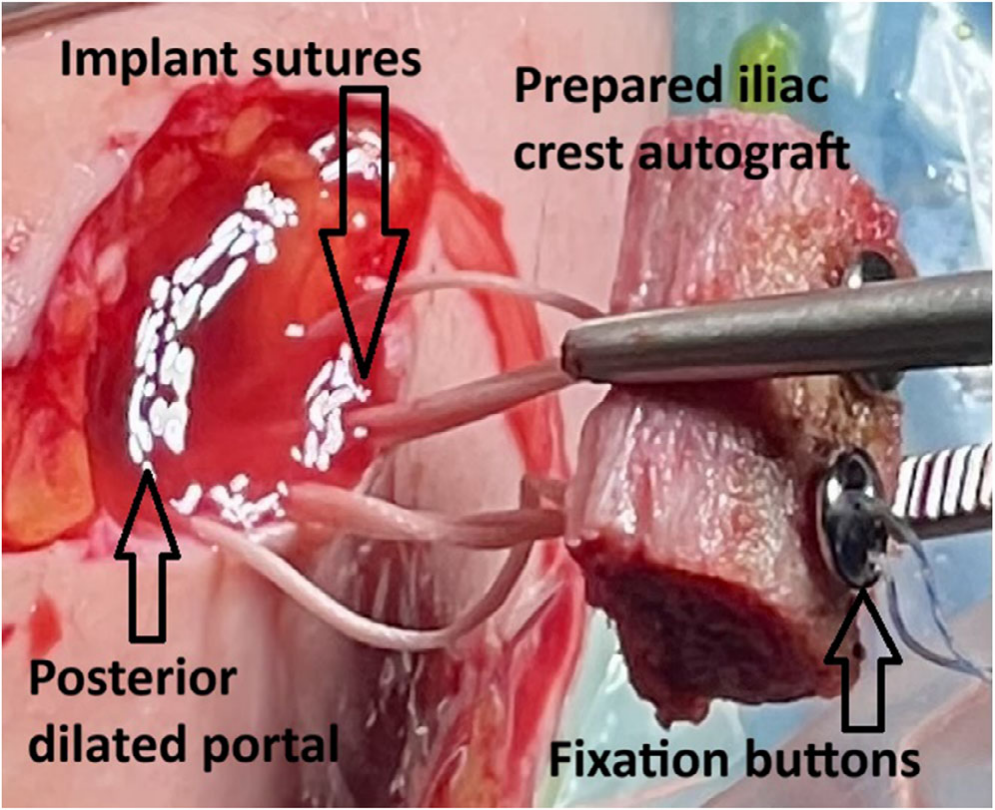
Placement of Smith & Nephew (Andover, MA, USA) cortical buttons on the cortical surface of the iliac crest autograft in preparation for fixation. The graft is introduced through the dilated posterior portal while traction is applied to the shuttle sutures from the anterior portal. A grasper introduced through the posterior portal assists smooth graft passage and correct orientation against the posterior glenoid.

The shuttle sutures are connected to the implant sutures, and the graft is introduced through the dilated posterior portal by pulling the sutures from the anterior portal (Figure 9). A grasper introduced through the posterior portal assists graft passage and orientation.

**FIGURE 10:**
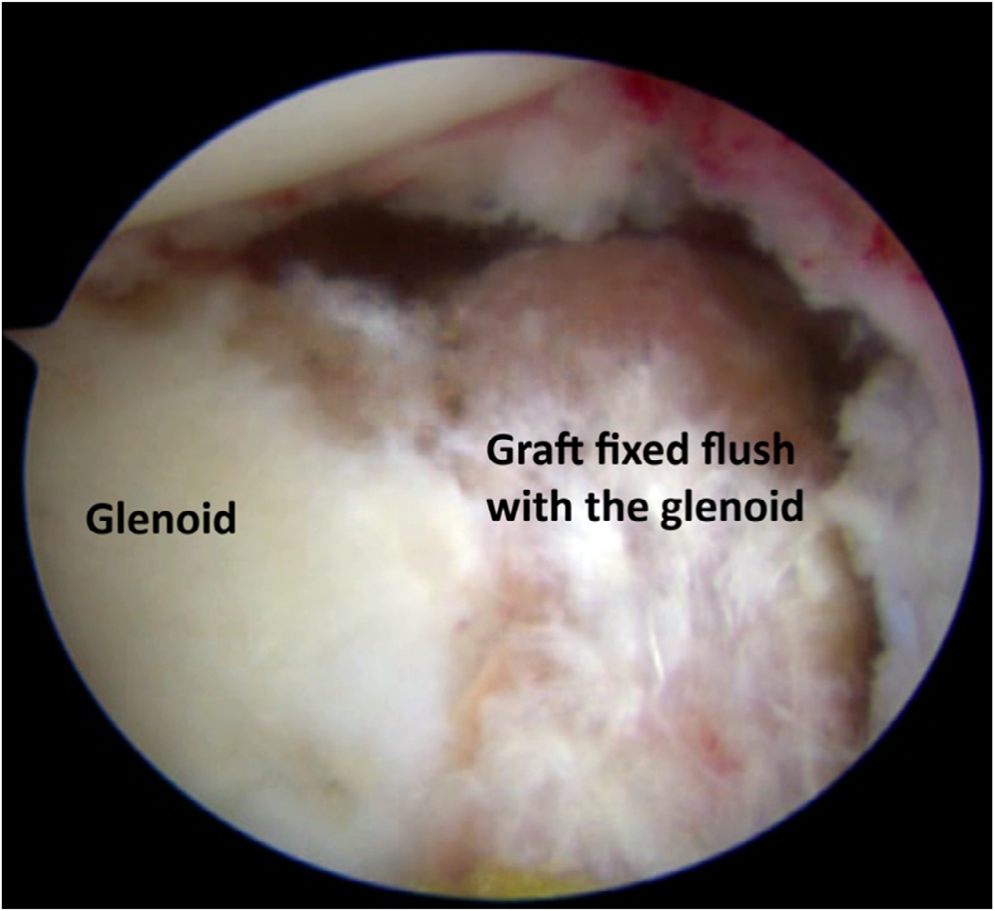
Arthroscopic view from the anterosuperior portal showing the final position of the iliac crest bone graft flush with the posterior glenoid surface. The graft is securely fixed using four cortical buttons (2 anterior and 2 posterior), providing strong, stable fixation and restoring posterior glenoid concavity without intra‐articular hardware prominence.

The graft is gently seated against the prepared posterior glenoid surface under direct visualization (Figure 10).

### Graft Fixation and Tensioning

Anterior cortical buttons are placed on the anterior glenoid cortex, and a Nice knot—a self‐locking sliding knot that allows progressive and controlled tensioning—is tied and advanced through the anterior portal.

Before final tensioning, the graft is carefully adjusted to sit flush with the glenoid articular surface, avoiding medialization or lateral overhang.

Final tensioning is performed using a calibrated tensioner to 100 N, repeated 3 times for each implant to ensure secure fixation, followed by additional security knots

### Posterior Capsulolabral Reconstruction

A meticulous posterior capsulolabral reconstruction is performed using 3 1.9‐mm all‐suture anchors (SutureFix; Smith & Nephew, Andover, MA), each loaded with double sutures.

**FIGURE 11:**
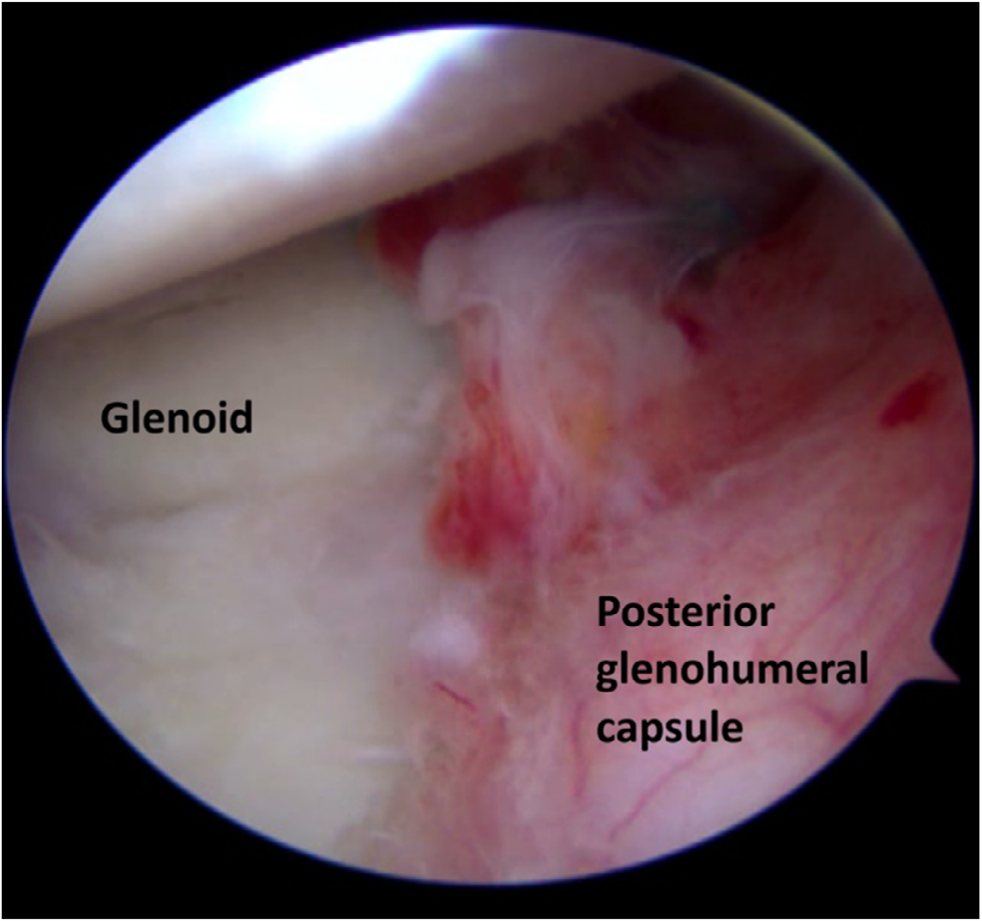
Final arthroscopic view from the anterosuperior portal after completion of posterior capsulolabral reconstruction using 3 all‐suture anchors. The posterior capsulolabral complex is restored, enhancing joint stability and proprioception, while the bone graft remains in an extra‐articular position, minimizing the risk of cartilage damage and postoperative osteoarthritis.

This reconstruction enhances joint stability, improves proprioception, and leaves the bone graft in an extra‐articular position, potentially reducing the risk of postoperative osteoarthritis (Figure 11).

### Final Assessment and Closure

Final assessment confirms graft stability, appropriate positioning, full range of motion, and absence of hardware prominence. Portals and the iliac crest donor site are closed in a standard fashion.

### Postoperative Rehabilitation

**FIGURE 12:**
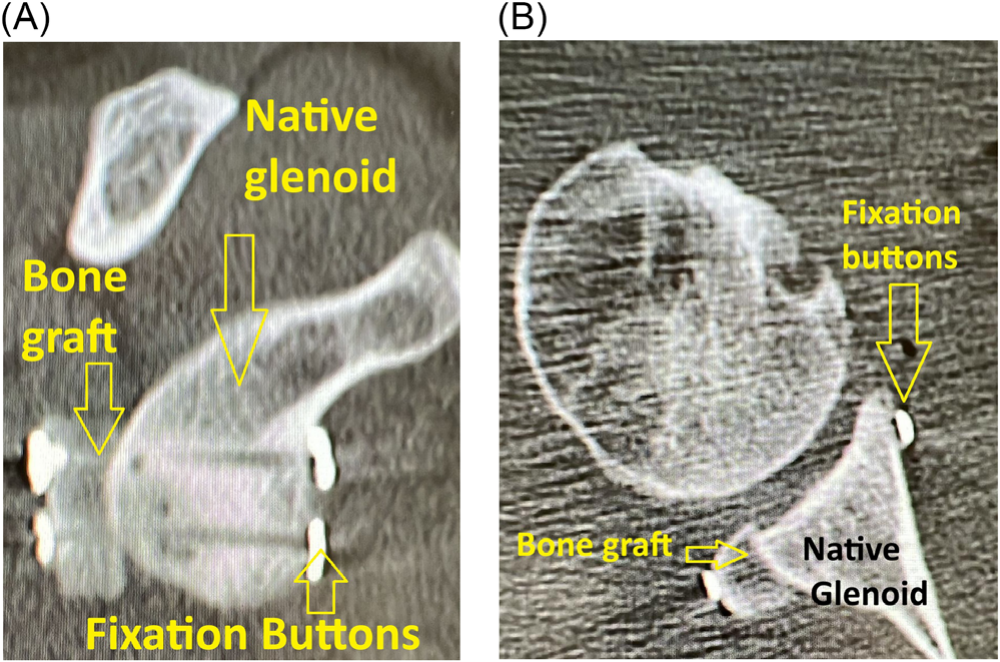
(A,B) Immediate postoperative computed tomography (CT) scans showing correct positioning of the posterior iliac crest bone graft flush with the glenoid surface and with a large bone‐to‐bone contact area. Stable fixation with cortical buttons and the use of an autograft optimize conditions for graft incorporation and long‐term restoration of posterior glenoid bone stock.

The shoulder is immobilized in neutral rotation for 4 weeks. Passive range of motion begins at 2 weeks, avoiding internal rotation and posterior loading. Active‐assisted motion is initiated at 4 to 6 weeks, followed by strengthening at 8 to 10 weeks. Return to contact sports is typically allowed after 5 to 6 months, depending on clinical and radiographic progression. A postoperative computed tomography scan is performed to check the correct position of the graft in relation to the glenoid (Figures 12A,B).

## DISCUSSION

Arthroscopic posterior bone block techniques have evolved substantially over the last decade.^5^, ^6^, ^7^, ^14^, ^15^, ^16^, ^17^, ^18^ Compared with open procedures, arthroscopy offers superior visualization, reduced soft‐tissue morbidity, and the ability to treat concomitant intra‐articular pathology.^13^, ^14^, ^15^, ^16^, ^17^

Independent cortical button fixation provides several advantages over screws and cerclage techniques.^5^, ^6^, ^7^, ^16^ It enables controlled compression, fine positional adjustment, and avoids complications such as hardware impingement, loosening, breakage, and secondary removal commonly reported with screws^19^, ^20^, ^21^, ^22^ (Table 2). Previous studies have reported graft resorption and osteolysis associated with rigid screw fixation.^11^, ^21^, ^23^, ^24^, ^25^

**TABLE 2:** Advantages and Disadvantages

**Advantages**	**Disadvantages**
Fully arthroscopic and minimally invasive	Technically demanding with learning curve
Direct visualization of tunnel and graft positioning	Requires specific instrumentation
Avoids screw‐related complications	Iliac crest donor‐site morbidity
Allows treatment of associated intra‐articular pathology	

The adapted use of an anterior glenoid guide improves reproducibility and shortens the learning curve, as most surgeons are familiar with this instrumentation from anterior instability procedures.^7^, ^16^ Our experience with iliac crest autograft in anterior bone block procedures has shown excellent integration and clinical outcomes, further supporting its use posteriorly.^16^, ^26^

Posterior instability remains a heterogeneous pathology with complex biomechanical and neuromuscular components.^17^, ^18^, ^19^, ^20^, ^21^, ^22^, ^23^, ^24^, ^25^, ^26^, ^27^, ^28^, ^29^, ^30^ Careful patient selection is essential. Although isolated soft‐tissue repairs may suffice in selected cases, bone block augmentation is indicated in the presence of structural deficiency, failed repairs, or glenoid dysplasia.^24^, ^25^, ^26^, ^27^, ^28^, ^29^, ^30^

Despite the advantages of arthroscopic posterior glenoid bone block augmentation, this technique is not exempt from risks and limitations. First, it presents a significant learning curve, as it requires advanced arthroscopic skills, familiarity with posterior shoulder anatomy, and precise control of graft orientation and fixation. Surgeons with limited experience in complex shoulder arthroscopy may encounter difficulties during graft passage, positioning, or fixation, potentially increasing operative time during the initial cases.

Second, the use of an iliac crest autograft introduces donor‐site morbidity, including postoperative pain, hematoma, sensory disturbances, and, rarely, fracture or infection. Although the tricortical graft harvested is relatively small, donor‐site complications remain an inherent disadvantage when compared with allograft‐based techniques.

Another limitation is the dependence on specific instrumentation, including a dedicated posterior glenoid guide and cortical button fixation system. Limited availability of these devices in certain institutions may restrict the widespread adoption of the technique. In addition, improper positioning of the guide or incorrect angulation during drilling may lead to malpositioned tunnels, risking insufficient graft compression, articular overhang, or penetration of the anterior cortex.

Tunnel‐related complications must also be considered. Creation of posterior‐to‐anterior glenoid tunnels carries a risk of iatrogenic glenoid fracture, tunnel convergence, or weakening of the glenoid vault, particularly in small glenoids or in cases with extensive bone loss. Careful preoperative planning and intraoperative fluoroscopic or arthroscopic control are therefore essential to minimize these risks.

Finally, long‐term outcomes regarding graft remodeling, resorption, and durability remain incompletely defined. Although short‐ and mid‐term clinical results of posterior bone block procedures are encouraging, further high‐quality studies with longer follow‐up are required to confirm the longevity of this reconstruction and its ability to prevent recurrent instability and degenerative changes.

In summary, the adapted use of a familiar anterior glenoid guide combined with independent cortical button fixation allows accurate, reproducible, and controlled posterior bone block placement while avoiding many of the complications associated with screw fixation and enabling comprehensive treatment of associated intra‐articular pathology.

## DISCLOSURES

The authors (P.C.S.P., M.I.P.R.) declare that they have no known competing financial interests or personal relationships that could have appeared to influence the work reported in this article.
